# Fluorescence Detection of Pb^2+^ in Environmental Water Using Biomass Carbon Quantum Dots Modified with Acetamide-Glycolic Acid Deep Eutectic Solvent

**DOI:** 10.3390/molecules29071662

**Published:** 2024-04-07

**Authors:** Shiwen Xing, Keyang Zheng, Lei Shi, Kaiming Kang, Zhixiao Peng, Xiaojie Zhang, Baoyou Liu, Huilong Yang, Gang Yue

**Affiliations:** 1 School of Environmental Science and Engineering, Hebei University of Science and Technology, Shijiazhuang 050018, China; 2 Hebei Key Laboratory of Pollution Prevention Biotechnology, Shijiazhuang 050018, China; 3 School of Food Science and Biology, Hebei University of Science and Technology, Shijiazhuang 050018, China

**Keywords:** ionic liquids, biomass carbon quantum dots, fluorescent probe, heavy metal monitoring, environmental monitoring, Pb^2+^

## Abstract

In this study, a novel green fluorescent probe material, nitrogen-doped carbon quantum dots (N-CQDs), was prepared by a one-step hydrothermal synthesis method using walnut green skin as a carbon source and acetamide-glycolic acid deep eutectic solvent (AGADES) as a modifier. By covalent coupling, the amide chromophore in AGADES is designed to cover the surface of walnut green skin carbon quantum dots (W-CQDs), forming a fluorescence energy resonance effect and improving the fluorescence performance of the carbon quantum dots. The prepared N-CQDs have a uniform particle size distribution, and the fluorescence quantum efficiency has increased from 12.5% to 32.5%. Within the concentration range of 0.01~1000 μmol/L of Pb^2+^, the linear detection limit is 1.55 nmol/L, which can meet the trace detection of Pb^2+^ in the water environment, and the recycling rate reaches 97%. This method has been successfully applied to the fluorescence detection and reuse of Pb^2+^ in actual water bodies, providing new ideas and methods for the detection of heavy metal ions in environmental water.

## 1. Introduction

Carbon quantum dots (CQDs), a new type of nanomaterial, are usually nearly spherical in shape and under 10 nm in size [1]. Over the past decade or so, CQDs have received increasing attention for their excellent properties, including good fluorescence properties, water solubility, biocompatibility, and low toxicity [2,3,4]. Based on these properties, CQDs are widely used in chemical sensing [5,6,7], catalysts [8,9], drug carriers [10], cellular imaging [11,12,13], and many more. In particular, based on their powerful and tunable photoluminescence properties, CQDs are effectively used for the detection of heavy metal ions [14,15,16], and the sensitivity and efficiency of the detection performance have been continuously innovated and explored, such as in elemental doping [17] and semiconductor compounds [18].

Since the surface functional groups of CQDs and the degree of oxidation are important factors affecting the surface energy level and energy gap, doping is an effective method to modify the functional groups on the surface of CQDs. By introducing local state functional groups, they are wrapped around the surface of CQDs, changing their electron transfer properties and forming a fluorescence energy resonance effect. This will improve the fluorescence quantum efficiency and sensitivity of CQDs, enhance their fluorescence performance, and expand their applications in different fields such as catalysis, energy science, and sensors [19]. Neha [20] et al. used amino acid salts to modify graphene carbon quantum dots and were able to improve the sensitivity and reduce the detection limit of carbon quantum dots. Varsha [21] et al. used paper as a carbon source and used APTES to pre-synthetically modify the paper precursors to synthesize CQD_S_ bioprobes with amine functional groups on the surface to improve the efficiency of binding to DNA. The work of other researchers has also demonstrated that surface passivation is an effective way to improve the fluorescence performance and application of CQDs.

Ionic liquids are often used as modified solvents to modify CQDs, but with the discovery of a new type of ionic liquid compound with a more complex supramolecular structure that has similar properties to ionic liquids, such as high density, low vapor pressure, and chemical stability, it is known as a “deep eutectic solvent (DES)”. The complexation of quaternary ammonium salts with hydrogen bond donors (e.g., acids, alcohols, amines, etc.) will result in charge separation between the anion and the hydrogen bond donor compound through oxygen bonding [22]. This will result in the formation of a low-eutectic mixture, which will lower the melting point. Low-eutectic solvents have attracted the attention of researchers because of their physicochemical similarity to ionic liquids, the simplicity of the preparation process, and the low cost of raw materials. 2003 saw the first study of the DES behavior of mixtures of urea or other amide derivatives with choline chloride (CC1) by Abbott [23] et al. DES can be prepared by simple physical mixing and heating or by freeze-drying the aqueous solutions of the components. preparation. This field is now developing rapidly, and DES has been used for a variety of purposes, such as microextraction solvents [24,25], solvents and catalysts in the synthesis of organics or polymers [26,27], and the trapping of CO_2_ [28,29]. DES can be used not only directly but also indirectly as an auxiliary material; e.g., Phosiri [30] et al. used DES-modified magnetic layered double hydroxides as sorbents to enrich organochlorine pesticides in environmental samples.

Lead ion (Pb^2+^) is a widely known chemical contaminant that has a significant impact on human health and the environment [31,32]. The World Health Organization (WHO) has established a maximum acceptable level of 50 ug/L (0.2413 μmol/L) for Pb^2+^ in drinking water and a maximum permissible level of 0.1 mg/L (0.4826 μmol/L) in surface water. Current methods for the detection of Pb^2+^, such as atomic absorption spectrometry [33], inductively coupled plasma mass spectrometry [34], and electrochemical methods [35], have low detection limits and high sensitivity and are widely used [36]. However, they have high requirements for instrumentation and equipment. Combining the research methods of CQDs and DES modification studies, it is feasible to develop a simple, efficient, green, highly sensitive, and selective method for the detection of lead ions.

In this paper, W-CQDs are prepared using biomass, reflecting the reuse of resources, and N-CQDs are prepared by using AGADES as a modifier. Through covalent coupling, the amide color-emitting group in AGADES is designed to cover the surface of W-CQDs, and the two form a fluorescence energy resonance effect to improve the fluorescence performance of N-CQDs, enhance the interaction between chemical substances and the surface of N-CQDs, optimize the structure of N-CQDs, and design different types of fluorescent probes according to needs, providing an alternative solution for the detection of heavy metals (Pb^2+^) in the environment.

## 2. Result

### 2.1. Structural Characterization of AGADES

**Figure 1:**
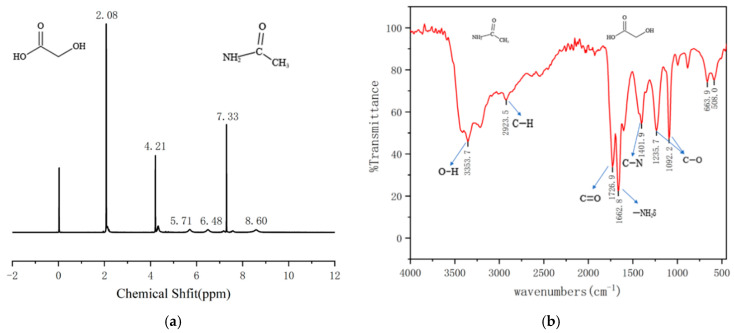

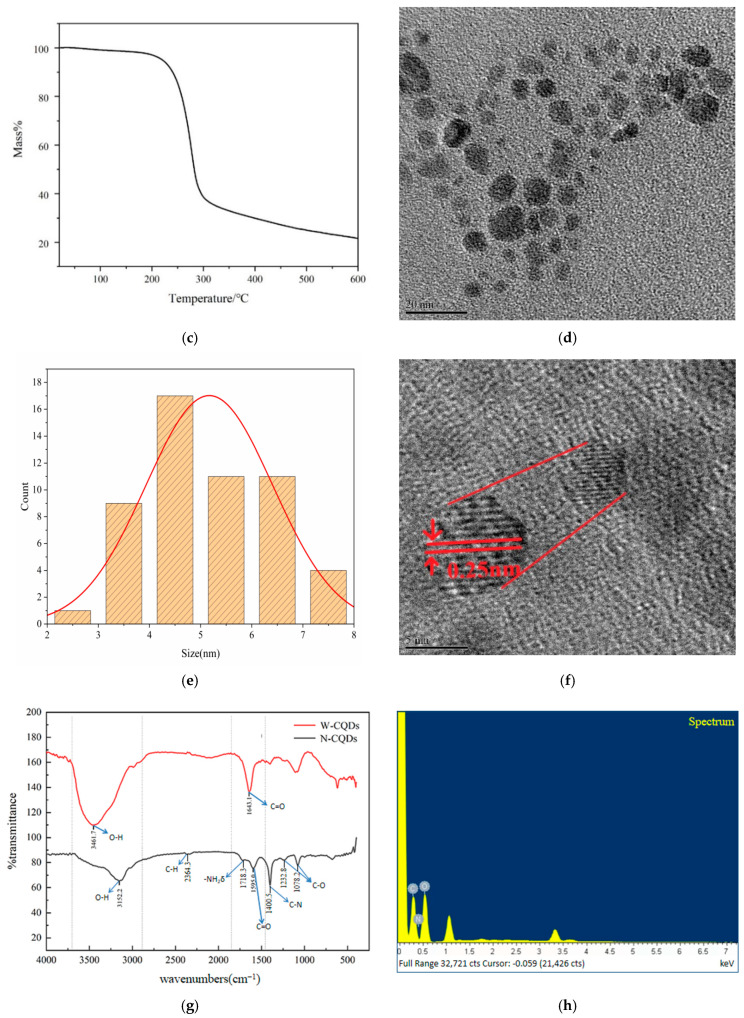

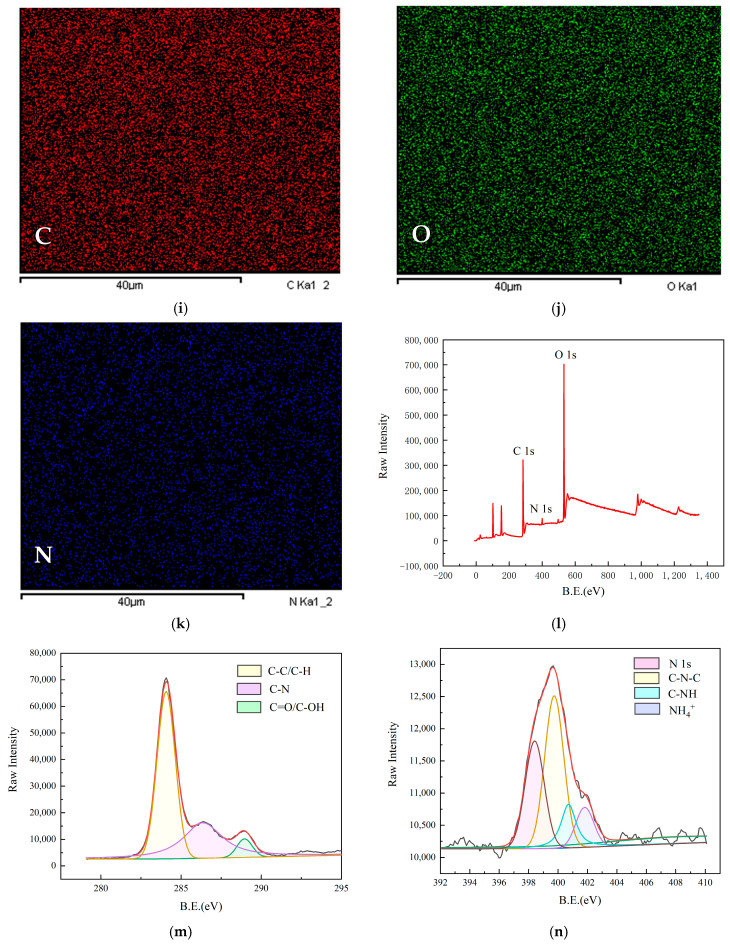

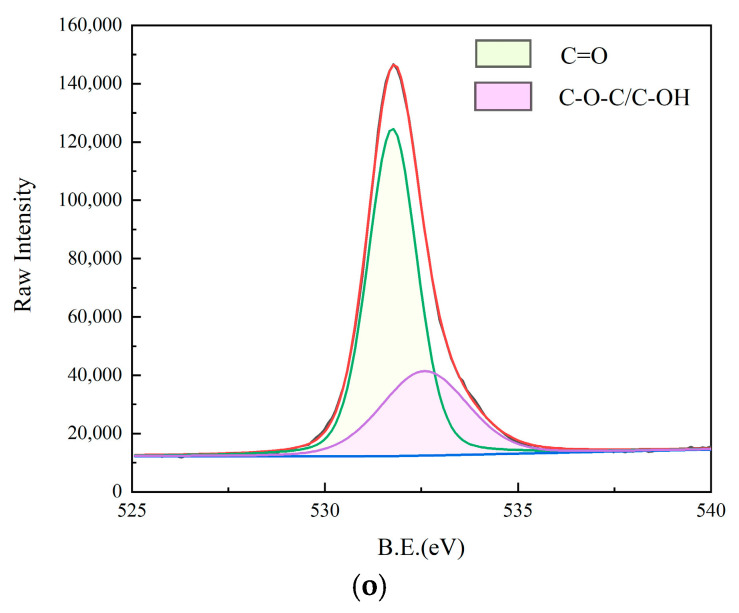
(**a**) is the ^1^H NMR of AGADES; (**b**) is the FT-IR plot of AGADES; (**c**) is the thermogravimetric schematic of AGADES; (**d**) shows the TEM image of N-CQD; (**e**) the particle size distribution: The column represents the number of carbon dots with different particle sizes in region Figure 1d, and the red line represents the normal distribution curve fitted according to the column chart; (**f**) the HRTEM image of the lattice structure of N-CQDs; (**g**) shows the FT-IR comparison between W-CQDs and N-CQDs; (**h**) shows the FT-IR of N-CQDs; (**i**–**k**) show the total EDS spectra, carbon, oxygen, and nitrogen spectra of N-CQDs, respectively; (**l**) shows the full spectrum scanning XPS spectra of N-CQDs; (**m**–**o**) shows the high resolution C1s spectra, N1s spectra, and O1s spectra of N-CQDs, respectively.

In order to investigate the structure of the N-CQDs, the specific structural composition of the modifier AGADES needed to be clarified first by performing ^1^H NMR and FT-IR measurements. The ^1^H NMR of the AGADES, shown in Figure 1a, revealed the presence of the -CH_3_ group at 2.08 ppm, a wave peak at 4.21 ppm indicating the presence of the -CH_2_ group, and a wave peak at a chemical shift of 5.71 ppm indicating the presence of the -OH group, according to the analysis of the ^1^H NMR spectrum of the AGADES. The wave peak at a chemical shift of 6.48 ppm indicates the presence of the -NH_2_ group. The wave peak at a chemical shift of 8.60 ppm is the -COOH group. Based on the comparison of the above characterization results with the results of the ^1^H NMR spectra analysis of acetamide and glycolic acid, it can be speculated that the reaction of the amide group of acetamide and the carboxyl group of glycolic acid during the reaction of acetamide and glycolic acid to form a DES causes a change in the density of the electrons and electron clouds outside the nucleus of the amide and carboxyl groups in the acetamide-glycolic acid molecule, which has a remote 1H chemical shift for the studied shielding effect on the studied 1H chemical shifts, which is why the above mentioned -NH_2_ groups and -COOH groups ^1^H NMR chemical shifts [37].

FT-IR characterization was performed to further confirm the chemical structure of AGADES, and the results are shown in Figure 1b. The broader telescopic vibrational absorption peak at 3353.7 cm^−1^ indicates the presence of the -OH group; the multiple vibrational peak at 2923.5 cm^−1^ indicates the presence of the -C-H structure; the absorption peak at 1662.8 cm^−1^ is the telescopic vibrational peak of the amide group, indicating the presence of the NH_2_ -C=O structure; the absorption peak at 1726.9 cm^−1^ is the telescopic vibrational absorption peak of the carbonyl group, indicating the presence of the C=O structure. The absorption peak at 1662.8 cm^−1^ indicates the presence of a C=O structure; the absorption peak at 1401.9 cm^−1^ indicates the presence of a C-N single bond; 1235.7 cm^−1^ and 1092.2 cm^−1^ are symmetrical duplex absorption peaks of C-O. The results of these analyses indicate that the main functional groups and structures of the substances measured are consistent with the prepared AGADES.

Thermogravimetric analysis of AGADES, as shown in Figure 1c, shows that AGADES has only a 3% reduction in mass until the temperature reaches 230 °C. The mass decreases sharply at 230 °C and by 62% at 300 °C. This indicates that the AGADES is able to maintain a relatively stable structural mass until 230 °C, which can satisfy the subsequent preparation of carbon quantum dots at 200 °C, and that the chemical structure of the AGADES substance remains unchanged.

### 2.2. Properties of CQDs Prepared on the Basis of AGADES

DES is a soft material formed by a 1:1 molar ratio of acetamide and glycolic acid, and its properties are different from the two materials themselves. It is by no means a simple superposition of the properties of the two substances. In order to investigate the structure and morphology of the prepared N-CQDs, a series of characterizations were carried out, including TEM, HRTEM, FT-IR, EDS, and XPS. TEM, Figure 1d,e of the N-CQDs show that the N-CQDs are approximately circular and spherical with a uniform distribution and an average particle size of approximately 4.67 nm. According to the HRTEM results, it can be observed that the crystalline surface spacing is 0.25 nm (Figure 1f).

The FT-IR comparison between W-CQDs and N-CQDs in Figure 1g shows a shift in the absorption peak of the O-H group at 3461.7 cm^−1^ and the absorption peak of the C=O group at 1643.1 cm^−1^. This is because the prepared N-CQDs are more prone to intermolecular hydrogen bonding between carbonyl and hydroxyl groups, which weakens the electron cloud density of carbonyl and hydroxyl groups and causes a red shift. In addition, it can also be seen from the comparison graph that N-CQDs have a stronger absorption peak for C-N bonds at 1400.5 cm^−1^, while W-CQDs do not, suggesting that the nitrogen is perfectly doped into the W-CQDs. Doping nitrogen atoms in W-CQDs by this method can cause radiative rearrangement, creating new surface state energy levels and electronic energy bonding through radiative rearrangement, thus improving the fluorescence efficiency of W-CQDs and the fluorescence performance of W-CQDs.

In addition, in Figure 1g, the FT-IR of N-CQDs shows the absorption peak at 2884.8 cm^−1^ for saturated C-H stretching vibration absorption and the asymmetric stretching vibration double peaks at 1232.8 cm^−1^ and 1078.2 cm^−1^ for C-O absorption. To verify the successful doping of the nitrogen atoms, the EDS results were used to plot and analyze the element types and atomic proportions of the N-CQDs. The concentration and atomic percentage of each element were 41.01%, 44.77%, and 14.22% for carbon, oxygen, and nitrogen atoms, respectively. The results of the surface scans are shown in Figure 1h–k, where the elements are uniformly distributed and the nitrogen atoms are successfully doped onto the surface of the N-CQDs at a high content, which is consistent with the FT-IR results.

XPS measurements were also performed to determine the chemical bonding between the elements. Figure 1l shows a full spectrum scan XPS spectrum with four different peaks at 284.1 eV, 400.1 eV, and 532.1 eV corresponding to C1s, N1s, and O1s. The high-resolution C1s spectrum in Figure 1m shows binding energies of about 284.1 eV, 286.4 eV, and 288.9 eV for the three peaks assigned to the C-C/C-H, C-N, and C=O/C-OH bonds; the high-resolution N1s spectrum in Figure 1n is integrally fitted to four sub-peaks with binding energies of 398.4 eV, 399.8 eV, 400.7 eV, and 401.9 eV for the four peaks of N1s, C-N-C, C-NH, and NH_4_^+^ bonds; the high-resolution O1s spectrum in Figure 1o shows two peaks with binding energies of about 531.8 eV and 532.7 eV, attributed to C=O and C-O-C/C-OH bonds, respectively.

In summary, the presence of a large number of hydrophilic groups on the surface of N-CQDs, such as O-H, N-H, C-O-C/C-OH, and C=O groups, gives N-CQDs excellent solubility and stability in water. The XPS results show the presence of amide groups in the prepared N-CQDs and confirm the successful doping of nitrogen atoms by the introduction of amide functional groups onto the surface of the W-CQDs by means of covalent coupling (amidation reaction) in an AGADES.

### 2.3. Optical Properties of N-CQDs

**Figure 2:**
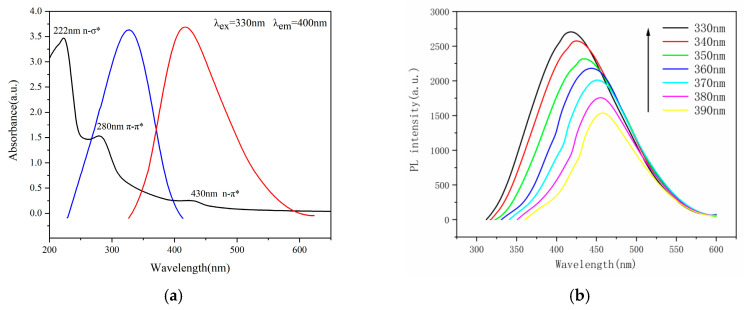

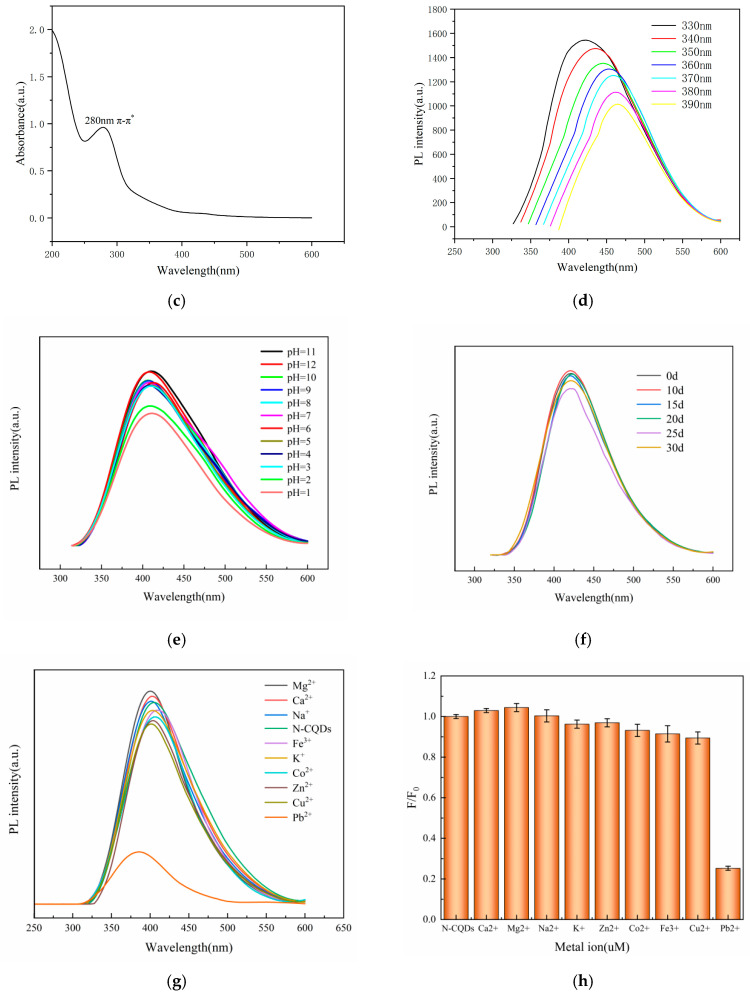

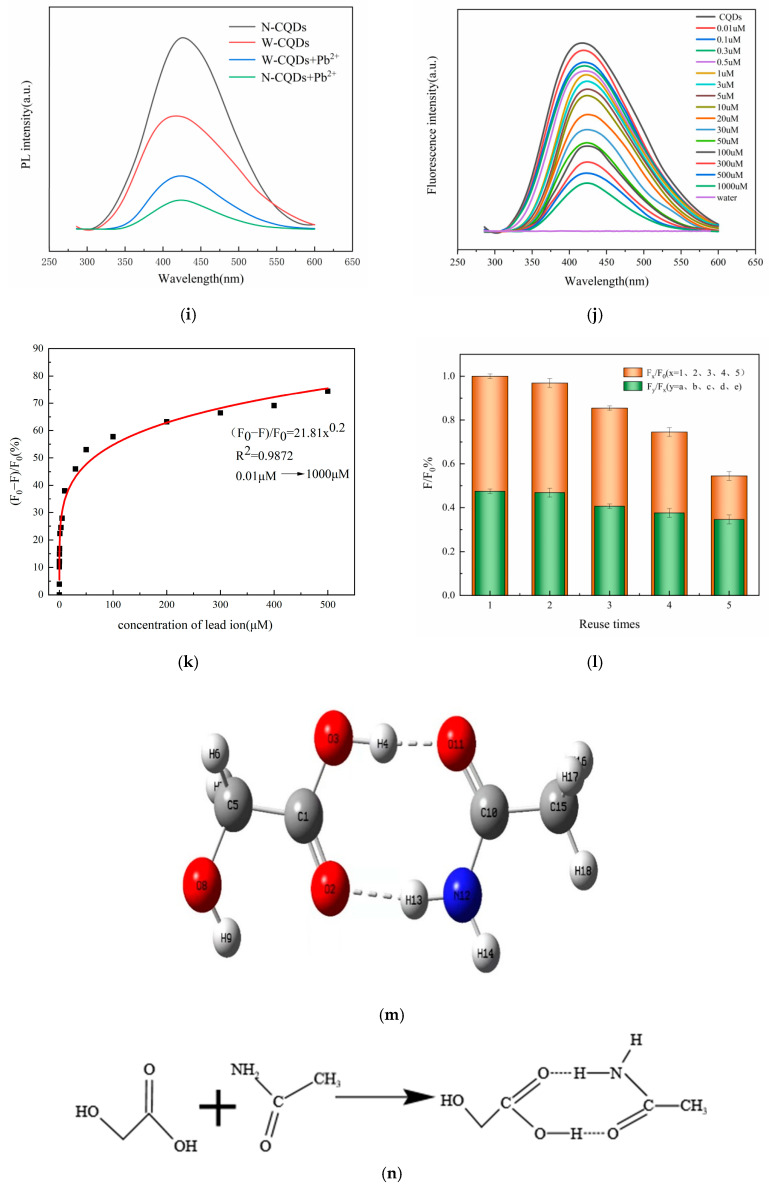

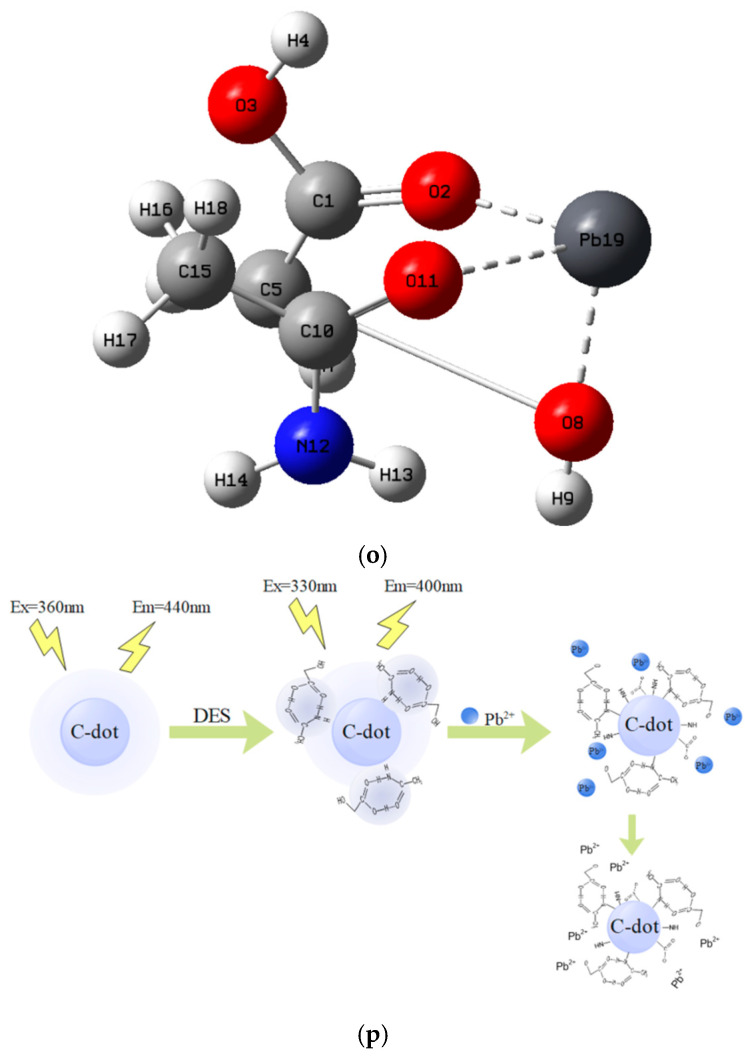
(**a**) UV–Vis spectra and fluorescence emission and excitation spectra of N-CQDs; (**b**) fluorescence emission spectra of N-CQDs at different excitation wavelengths from 330 to 390 nm; (**c**) UV–Vis spectra of W-CQDs; (**d**) fluorescence emission spectra of W-CQDs at different excitation wavelengths from 330 to 390 nm; (**e**) fluorescence spectra at different pH; (**f**) fluorescence spectra of N-CQDs at different times; (**g**) Effect of different metal ions on the fluorescence intensity of N-CQDs at 330 nm excitation wavelength; (**h**) Fluorescence burst efficiency of N-CQDs by different metal ions; (**i**) Comparison of the effect of N-CQDs and W-CQDs with Pb^2+^; (**j**) Fluorescence spectra of N-CQDs in response to different concentrations of Pb^2+^; (**k**) Linear relationship between Pb^2+^ concentration and the fluorescence quenching rate of N-CQDs (F_0_−F)\F_0_; (**l**) shows the efficiency of N-CQDs recycled five times. (**m**) shows the molecular structure simulation of AGADES; (**n**) shows the reaction process equation of AGADES; (**o**) The optimized cluster structure of ACE:GA:Pb (1:1:1) has a bond length of O11···Pb19 = 2.5065; O2···Pb19 = 2.0554; O8···Pb19 = 2.0223; (**p**) Fluorescence quenching mechanism of N-CQDs by lead ions.

In the UV–Vis spectrum of N-CQDs (black line), Figure 2a, the different absorption peaks are concentrated at 222 nm, 280 nm, and 430 nm, with one absorption peak at 222 nm attributed to an n-σ* leap, indicating the presence of -NH_2_. The absorption peak at 280 nm can be attributed to a π-π* leap in the aromatic sp2 structural domain and has strong absorption, including C=C and C=O bonds. The C=O bond can also undergo an n-π* leap, which requires less energy and can have less strong absorption in the near-UV or visible region, while the absorption peak at 430 nm near the long-wave region is attributed to a red shift phenomenon where the maximum absorption peak is shifted towards the long-wave direction due to the influence of the substituent or solvent [38]. Polar solvents can shift the UV absorption peak in the long-wavelength direction. The carbonyl and hydroxyl functional groups of CQDs will form hydrogen bonds, and the formation of hydrogen bonds will also shift the UV absorption peak towards the long wavelength direction. Under irradiation at the 330 nm excitation wavelength, N-CQDs emit a maximum of 400 nm (red line) and emit blue light in the UV. Fluorescence emission spectra of N-CQDs at different excitation wavelengths from 330 nm to 390 nm, Figure 2b.

Figure 2c shows the UV–Vis spectrum of W-CQDs, and Figure 2d shows the fluorescence spectrum of W-CQDs. By comparing Figure 2a,c, it can be observed that the fluorescence intensity changes uniformly at different excitation wavelengths, and both reach their optimal excitation wavelength at 330 nm, while N-CQDs exhibit stronger fluorescence intensity at the same excitation wavelength. By comparing Figure 2b,d, it can be observed that due to the doping of AGADES, it increases the n-σ* and n-π * transitions. It is speculated that the formation of -N-H and -C=N- structures on the surface of carbon dots is the cause.

The fluorescence quantum yield of N-CQDs was calculated at the optimal excitation wavelength of 330 nm. Quinine sulfate solution was prepared by dissolving quinine sulfate in 0.1 mol/L H_2_ SO_4_ (Φ_s_ = 54%, 360 nm) as a standard, and the fluorescence quantum yield was calculated according to Equation (1).

(1) $$\Phi_{R}=\Phi_{S}\times \frac{F_{S}}{F_{R}}\times \frac{A_{R}}{A_{S}}\times {\left(\frac{n_{S}}{n_{R}}\right)}^{2}$$

Φ_s_ and Φ_R_—are the fluorescence quantum yields of the samples and the fluorescence quantum yields of quinine sulfate, respectively;

*F_S_* and *F_R_*—are the integrated fluorescence intensities of the sample and quinine sulfate, respectively;

*A_S_* and *A_R_*—are the absorbances of the solutions at the excitation wavelengths of the sample and quinine sulfate respectively (to minimize the reabsorption effect, the absorbance values of the solutions measured should both be less than 0.05);

*η_S_* and *η_R_*—are the refractive indices of the sample and quinine sulfate, respectively.

The fluorescence quantum efficiency of N-CQDs was calculated to be 32.5%, while that of W-CQDs was 12.6%. This indicates that through the modification of DES, nitrogen-containing organic molecules effectively passivate the surface of carbon dots and improve the fluorescence quantum yield of carbon dots.

### 2.4. Effect of Reaction pH on N-CQDs

The sensitivity of Pb^+^ sensing is determined by the specific selective reaction of N-CQDs and is not affected by pH values. To investigate the effect of pH on the N-CQDs, the pH was adjusted to 1, 2, 3, 4, 5, 6, 7, 8, 9, 10, 11, and 12 with NaOH and HCl at a concentration of 0.1 mol/L. The results of the fluorescence analysis are shown in Figure 2e. The pH of the original N-CQDs was measured to be weakly basic at 8.18 using a pH indicator. This was due to the amidation reaction between the carboxyl groups contained on the surface of the W-CQDs and the acyl compounds in the modifier AGADES, which condensed the amide groups onto the surface of the W-CQDs through a standard EDC/NHS coupling reaction, successfully doping the nitrogen into the W-CQDs to produce N-CQDs. When pH = 1 and pH = 2, the fluorescence intensity of N-CQDs decreases more significantly as the solution becomes more acidic, due to the hydrolysis of the amino and amide groups on the surface of N-CQDs in a strongly acidic environment [39]. The fluorescence performance of N-CQDs did not change significantly under other pH conditions, indicating that N-CQDs are pH-adapted and can be used in weakly acidic, alkaline, and neutral environments with good pH stability, providing a favorable use for subsequent assays without additional pH adjustment.

### 2.5. Temporal Stability of N-CQDs

To investigate the effect of time on N-CQDs, N-CQDs were placed for 0, 5, 8, 12, 15, 25, and 30 days, and their fluorescence stability was monitored over a period of one month, as shown in Figure 2f. There was a slight and irregular overall decrease in fluorescence intensity, which may be related to the aggregation of N-CQDs over a long period of time.

### 2.6. Specific selectivity of N-CQDs for Pb^2+^

The emission spectra of N-CQDs for nine different metal ion solutions, as shown in Figure 2g, revealed that the response values of all metal ions to N-CQDs except Pb^2+^ were not high and the fluorescence response rates were less than 10%, while the fluorescence response values for Pb^2+^ reached 86%, as shown in Figure 2h, reflecting the high selectivity and sensitivity of N-CQDs to Pb^2+^ and the strong anti-interference ability. Furthermore, it is worth noting that the fluorescence intensity of these nine metal ion solutions is close to zero at the same excitation wavelength and test conditions. Therefore, the fluorescence from the ambient background does not interfere with the application of N-CQDs, and the experimental results of this study are reliable. Figure 2i shows the comparison of the responsiveness of W-CQDs and N-CQDs to Pb^2+^, and it is found that the W-CQDs are 45%, while the N-CQDs are 92%, which is twice as high as the W-CQDs, indicating that the sensitivity to Pb^2+^ is greatly enhanced due to the doping of N-CQDs with N elements.

Figure 2j shows the emission spectra of the obtained N-CQDs in different concentrations of Pb^2+^ solution. The fluorescence intensity of the N-CQDs gradually decreased with increasing Pb^2+^ concentrations. As shown in Figure 2k, the fluorescence response rate of N-CQDs (F_0_−F)/F_0_ was fitted with Pb^2+^ concentration in the concentration range of 0.01–1000 μm, which matched the exponential correlation (F_0_−F)/F_0_ = 21.81x^0.2^ and the correlation coefficient squared (R^2^) = 0.9872, from which the LOD = 1.55 nmol/L (LOD = 3 δ/S). The above detection limits demonstrate the wide detection range of the method, which is lower than the drinking water standard of 0.2413 μmol/L and the surface water standard of 0.4826 μmol/L, indicating that N-CQDs can achieve efficient trace detection of Pb^2+^ in water.

### 2.7. Detection of Pb^2+^ in Actual Water Samples

To evaluate the practicality and feasibility of this method, laboratory tap water and river water from the Hebei University of Science and Technology campus were collected and tested. Prior to the experiment, the water samples were filtered through 0.22 µm microporous filter heads to remove insoluble impurities, and different concentrations of Pb^2+^ standard solutions were added to the obtained water samples. The solutions were then added to the solutions in N-CQDs, respectively. The exact amount of Pb^2+^ was obtained by the standard addition method [40]. The results are shown in Table 1, and the recoveries of the standard addition method were 95.40–102.20% with relative standard deviations of 1.10–2.10%, indicating that the material can be successfully applied to the determination of Pb^2+^ in real water samples.

### 2.8. Recycling Experiments with N-CQDs

In order to investigate the recycling performance of N-CQDs, the fluorescence intensity of the original N-CQDs was recorded as F_0_, to which lead ions were added, mixed, and shaken to measure the fluorescence intensity. 0.2 g of sodium polyaspartate was added to the mixed solution of N-CQDs and lead ions, and after mixing and shaking, the phenomenon was left for 30 min to observe, and it was found that a flocculent precipitate appeared in the mixed liquid, which was due to the chelation of polyaspartic acid and Pb^2+^ [41]. Polyaspartic acid is stronger than the complexation of N-CQDs and Pb^2+^, and the flocculant precipitate is formed. By using high-speed centrifugation at 10,000 r/min to remove flocculent precipitates and extract the supernatant, N-CQDs can be recycled, reflecting the concept of economic sustainability. The N-CQDs were reused five times as described above, and the recycling rate (RR) and fluorescence quenching rate (FQR) of the N-CQDs were measured as in Equations (2) and (3) below.

(2) $$RR\left(\%\right)=\frac{F0}{Fx\left(x=0,\;\;\;2,\;\;\;3,\;\;\;4\right)},$$

(3) $$FQR\left(\%\right)=\frac{Fy}{Fx\left(y=a,\;\;\;b,\;\;\;c,\;\;\;d,\;\;\;e\right)}\;$$

In Equations (2) and (3)

*F*_0_, *F*_1_, *F*_2_, *F*_3_, *F*_4_—Fluorescence intensity of N-CQDs, N-CQDs-1, N-CQDs-2, N-CQDs-3, N-CQDs-4.

*F_a_*, *F_b_*, *F_c_*, *F_d_*, *F_e_*—corresponding fluorescence intensity of lead ions.

The results of the N-CQDs repeated use experiment are shown in Figure 2l. The decrease in fluorescence intensity of N-CQDs at the second and third times of repeated use was not significant, while the fluorescence intensity decreased to 59% at the fifth time, which was due to the decrease in the content of N-CQDs in the solution and the fluorescence intensity as a result of operations such as centrifugation and filtration during the repeated use. Although there were varying degrees of reduction in the fluorescence intensity of N-CQDs over five cycles, the response efficiency of N-CQDs to lead ions remained at 91–97%, indicating that N-CQDs can support at least five cycles of use with a recovery rate of 82–97% over five cycles, reflecting the stability and value of the use of N-CQDs.

### 2.9. Principle of Pb^2+^ Detection by N-CQDs

To further investigate the response mechanism of N-CQDs, the AGADES were simulated using Gaussian view and Gaussian 09W, and the chemical structure of the AGADES was simulated and the reaction equation was deduced [42]. The energy configuration of acetamide and glycolic acid was calculated in a 1:1 ratio at the level of B3LYP/6-31G. Geometric optimization was carried out to obtain the best molecular conformation with the lowest energy. The optimized molecules were imported into the Traj.xyz file, and in this environment, the emission, spin, and collisions between the monomers continued, resulting in the optimal cluster structure. In addition, 100 molecular clusters were generated under GENMER (acetamide:glycolic acid = 1:1), and structural optimization was performed in MOPAC at the PM DH+ exact level to obtain the lowest energy optimal molecular configuration as shown in Figure 2m. The analysis of the 1H NMR and FT-IR measurements of AGADES also verified that the quantum chemical calculations were in agreement with the experimental results. The inferred AGADES reaction process based on the AGADES structural formula is shown in Figure 2n.

The effective fluorescence quenching of N-CQD materials by Pb^2+^ is due to the complexation of Pb^2+^ with amino and carboxyl groups, resulting in the reconstruction of the structural framework of N-CQDs. The amino group in N-CQDs is the amide group grafted onto the surface of W-CQDs by AGADES through the amide reaction by covalent coupling, successfully doping the nitrogen element into the W-CQDs to form N-CQDs, increasing the electron cloud density on the surface of N-CQDs, thereby increasing the conjugation system and enhancing the fluorescence performance of N-CQDs [43]. Gaussian simulation was performed to generate 100 molecular clusters (acetamide:glycolic acid:Pb^2+^ = 1:1:1) under GENMER, and structural optimization at the precise level of PM DH+ was performed in MOPAC to obtain the optimal molecular configuration with the lowest energy, as shown in Figure 2o. The abundant hydroxyl, carboxyl, and amino groups on the surface of the functionally designed N-CQDs will have a strong metal-ligand interaction with the metal Pb^2+^, and a complexation reaction occurs (Figure 2p of the principle). Thus, when Pb^2+^ is added to the N-CQDs solution, it will cause the complexation of the color-emitting groups hydroxyl, carboxyl, and amino groups on the surface of the N-CQDs with Pb^2+^, thus leading to different degrees of fluorescence burst of N-CQDs with good Pb^2+^ detection. Table 2 presents a comparison of different methods for the detection and treatment of Pb^2+^.

## 3. Materials and Methods

### 3.1. Materials and Instrumentation

Acetamide, glycolic acid, copper sulfate, magnesium sulfate, zinc chloride, lead nitrate, iron sulfate, sodium chloride, magnesium sulfate, calcium sulfate, potassium chloride, and walnut green peel (Y65-1-1). All reagents were analytically pure, and no further purification was required for use. Transmission electron microscopy (TEM) images were taken using an F20 TEM (Tokyo, Japan, Japan JEOL, JEM-2100) at an accelerating voltage of 200 kV; energy dispersive spectrometry (EDS) and mapping images were collected using an energy dispersive spectrometer (Karlsruhe, Germany, Bruker, Quantax XFlash series); X-ray photoelectron spectroscopy (XPS) was performed in standard lens mode (ThermoFischer, Waltham, MA, USA) using an ESCALAB 250Xi X-ray photoelectron spectrometer with Al K Alpha (hv = 1340 eV); nuclear magnetic resonance spectra were recorded using deuterated chloroform as solvent (Karlsruhe, Germany, Bruker, Avance 500 MHz) (^1^H NMR); fluorescence emission spectra were recorded using an F-7000 fluorescence spectrophotometer (Hitachi, Japan); a Tencer 27 Fourier transform infrared spectrometer from Bruker was chosen to characterise the infrared spectrum of DES; and ultraviolet and visible (UV–Vis) spectra were obtained using a UV-6100S UV–Vis spectrophotometer absorption spectra (Shanghai, China, Metash Instruments Ltd., UV-6100S).

### 3.2. Preparation of AGADES

The acetamide and glycolic acid were ground separately in a grinder and then ground thoroughly in a 1:1 molar ratio of acetamide and glycolic acid. The ground solid powder was added to a 50 mL round bottom flask and heated in a continuous water bath at a constant temperature of 363.15 K in a heat-collecting magnetic stirrer under nitrogen protection until a homogeneous, clear liquid was formed [48].

### 3.3. Preparation of N-CQDs and W-CQDs

Due to the fact that Hebei Province is one of the main producing areas of walnuts and the convenience of obtaining materials, the walnut green skin used in this study was collected from Pingshan soft walnuts from Hebei University of Science and Technology in August 2021. Using it as a biomass carbon source can avoid waste and make reasonable use of walnut green husk.

#### 3.3.1. Preparation of W-CQDs

Grind the selected walnut green skin with a grinder, pass through a 100 mesh sieve, take 0.1 g, and add 30 milliliters of water. Ultrasound oscillation at 12 kw for 30 min. After centrifugation, take 4 mL of supernatant and directly add 30 mL of water to a high-pressure vessel lined with polytetrafluoroethylene at 220 °C for 14 h. Subsequently, the obtained liquid was centrifuged at a high speed of 10,000 r/min and filtered 3–5 times using a 0.22 μm membrane. The supernatant was taken to obtain W-CQDs.

#### 3.3.2. Preparation of N-CQDs

**Figure 3:**
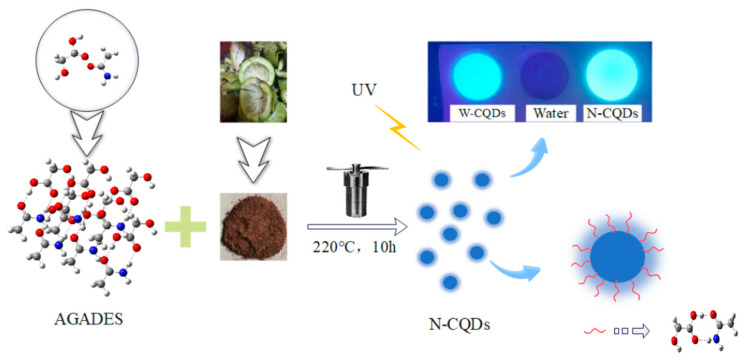
Experimental flow chart for N-CQDs.

According to the above steps, after centrifuging the supernatant, add 1 g of AGADES and 30 mL of water prepared above to a high-pressure vessel lined with polytetrafluoroethylene. For other steps, take the supernatant to obtain N-CQDs. The experimental flowchart of N-CQDs is shown in Figure 3.

### 3.4. Determination of the Fluorescence Stability Properties of N-CQDs

To assess the effect of time on the stability of the N-CQDs, fluorescence spectrophotometry was performed at 0, 3, 9, 14, 25, 30, 40, 54, and 60 d. The change in stability of the N-CQDs was analyzed over a period of two months. To assess the effect of pH on the stability of the N-CQDs, the N-CQDs were formulated with 0.1 mol/L NaOH and 0.1 mol/L HCl, and N-CQDs configured at different pHs (1–14) were monitored with a pH indicator and subsequently subjected to fluorescence spectrophotometry. All the above N-CQDs were determined by fluorescence analysis at an excitation wavelength of 330 nm.

### 3.5. Selective Determination of N-CQDs

The specific selectivity of N-CQDs for metal lead ions was determined by comparing the fluorescence burst response of N-CQDs to nine metal ions. A 10 mmol/L solution of Na^+^, Mg^2+^, Ca^2+^, Zn^2+^, Fe^3+^, K^+^, Pb^2+^, Cu^2+^, and Co^2+^ metal ions was prepared and mixed at a volume ratio of 1:1 (metal ions:N-CQDs) with ultrasonic shaking for 30 min, followed by fluorescence spectrophotometric determination.

## 4. Conclusions

This work uses the AGADES reagent to functionalize biomass carbon quantum dots, grafting the amide group in AGADES onto the surface of W-CQDs by covalent bonding through the amide reaction, and successfully doping nitrogen into the W-CQDs to produce N-CQDs. The fluorescence quantum yield of N-CQDs was increased from 12.6% to 32.4%. Spectroscopic studies have shown that N-CQDs have good photoluminescence, stability, and anti-interference properties. N-CQDs detect the heavy metal Pb^2+^ in water with good anti-interference properties and a response rate of 92%, twice that of W-CQDs 45%, which is due to the large chromophores (amide and carboxyl groups) on the surface of N-CQDs. The response of N-CQDs to Pb^2+^ corresponds to an exponential correlation in the range of 0.01 to 1000 uM, with a limit of detection (LOD) of 1.55 nmol/L, which is lower than the standard for domestic drinking water of 0.2413 μmol/L and can meet the application of trace detection of Pb^2+^.

In addition, the above N-CQDs were able to meet at least five recycling cycles with 82–97% recovery in recovery experiments. N-CQDs can be used for the detection of trace Pb^2+^ in water samples, which broadens the application of carbon quantum dots as potential future nanoprobes for metal ion detection.

## Figures and Tables

**Table 1:** Determination of Pb^2+^ concentrations and recoveries in actual water samples (*n* = 3).

Sample	Add/μmol/L	Found/μmol/L	Recovery/%
Ground water	5	5.1	102.2
	10	9.9	99.2
	15	14.5	96.3
Lake water	5	5.0	99.6
	10	9.5	95.4
	15	15.1	100.8

**Table 2:** Comparison of different methods for detection and treatment of Pb^2+^.

Fluorescent Sensors	Linear Range (μmol/L)	LOD (nmol/L)	Can It Be Recycled	Literature
Fluorescent carbon quantum dots	50–50,000	4.78	No	[44]
Fluorescent carbon quantum dots	0.0167–1	4.6	No	[45]
Fluorescent graphene quantum dots	600–50,000	470	Yes	[46]
Nanocomposite materials Au(NP)/TCODS/BN	4.8–290	3.9	No	[39]
Biomass carbon quantum dots	1.3–106.7	3.2	No	[47]
Fluorescent carbon quantum dots	0.01–1000	1.55	Yes	This work

## Data Availability

The data presented in this study are available on request from the corresponding author.
